# Development of Goal-Achievement Support App to Assist Children and Families in Participating in Meaningful Occupations: Content Validation Using Delphi Method

**DOI:** 10.2196/73430

**Published:** 2025-08-20

**Authors:** Koki Kura, Jumpei Oba, Satoru Amano, Kayoko Takahashi

**Affiliations:** 1 Department of Occupational Therapy, Faculty of Rehabilitation, Kobe Gakuin University, Kobe, Japan; 2 Graduate School of Medical Sciences, Kitasato University, 1-15-1, Kitasato, Minami-ku, Sagamihara, Kanagawa, 252-0373, Japan, 81 42 778 9305; 3 Occupational Therapy Course, Department of Rehabilitation, School of Allied Health Science, Kitasato University, Kanagawa, Japan

**Keywords:** children’s occupation support mobile system, home-centered interventions, neurodevelopmental disorders, pediatric occupational therapy, family-centered care, mobile health, mobile app, occupational therapy

## Abstract

**Background:**

Occupational therapy has highlighted the necessity for planning and executing interventions in collaboration with clients, families, and caregivers to facilitate their progress. Thus, in pediatric occupational therapy, it is essential to position the family as a primary client and to actively involve them in the intervention process. These interventions often incorporate tools that facilitate parental engagement in home-based activities. However, no tools have been specifically designed to support parents comprehensively in achieving their parenting goals in everyday situations. To address this gap, we developed a mobile app called the Children’s Occupation Support Mobile System (COSMO) to support occupational therapists, children, and parents in a collaborative manner to achieve intervention goals in daily life.

**Objective:**

The aim of the study is to develop the COSMO and validate its content in terms of legibility, visibility, and accessibility.

**Methods:**

This study was conducted in two stages: (1) designing a prototype of COSMO and (2) validating its content using the Delphi method. The prototype was developed based on a conceptual model of parenting strategies, which was derived from interviews with mothers raising children with developmental disabilities. This study included 10 Japanese pediatric occupational therapists, who were selected using convenience sampling to ensure diversity and heterogeneity in attributes. The Delphi survey was conducted entirely through a web-based questionnaire emailed to the experts. Participants rated their agreement with each item on a 5-point Likert scale. A mean item score of ≥3.75 (75%) indicated consensus.

**Results:**

The prototype was designed through a series of 13 one-hour meetings held monthly. The functional framework of COSMO was structured into four core components based on previous research: (1) collaborative goal setting, (2) home strategy—an action plan to achieve goals, (3) self-reflection—a record of implemented strategies, and (4) progress reports—data storage for tracking outcomes. For validating the content, the 2 Delphi rounds resulted in a total mean score of 4.44 for legibility, 4.86 for visibility, and 4.84 for accessibility. In the free-text responses, there were references to improvements in the wording and to the burden of writing the reflections. Therefore, the wording was revised to avoid jargon and use plain language. The burden of COSMO use was reduced by simplifying the use process by incorporating optional inputs for some of its functions.

**Conclusions:**

COSMO was developed as a comprehensive tool to integrate functions while aiming to reduce the burden on parents. This may reduce resistance to app use and make it easier for more parents to use it. Future studies should evaluate the generalizability and effectiveness of the prototype as an intervention. Limitations of this study include the absence of end-user testing, a geographically limited expert panel, and a limited discussion of implementation challenges across diverse health care settings.

## Introduction

The World Health Organization emphasizes the importance of designing interventions for children with neurodevelopmental disorders, including autism, through the active participation of all stakeholders [1]. Furthermore, these interventions should be implemented at the community and social levels to enhance accessibility, inclusivity, and support [1]. Similarly, the American Occupational Therapy Association (AOTA) highlighted the necessity for planning and executing occupational therapy interventions in collaboration with clients, families, and caregivers to facilitate their progress [2]. Thus, in pediatric occupational therapy, it is essential to position the family as a primary client and actively involve them in the intervention process.

A systematic review of pediatric occupational therapy interventions recommends an activity-focused top-down approach that prioritizes collaboration with caregivers [3]. Specifically, interventions should begin with child-centered goals, incorporate real-life activities in natural settings, and use intensive repetition and problem-solving at an appropriate difficulty level [3]. Furthermore, interventions should be seamlessly integrated into daily life environments, such as homes and schools, while ensuring active family and caregiver participation [4].

Therefore, pediatric occupational therapy should emphasize family engagement and practical activity-based support in natural living environments. Several occupational therapy interventions have adopted family-centered approaches in real-life settings. Examples include Occupational Performance Coaching [5], Home Programs [6], Cog-Fun [7], Pathways and Resources for Engagement and Participation [8], and Parental Occupational Executive Training [9]. These interventions often incorporate tools that facilitate parental engagement in home-based activities. For instance, intervention booklets outline objectives and policies [10], structured documents help record strategies [8], and diaries support tracking daily progress [9]. These tools assist in setting collaborative intervention goals, reinforcing strategies, and documenting progress. However, no tools have been specifically designed to fulfill these functions comprehensively and to support parents in achieving their parenting goals in everyday situations. Everyday situations refer to parenting behaviors that occur outside the occupational therapy room, where the occupational therapist cannot be involved. Examples include self-care activities such as eating and dressing, productive activities such as learning and playing at home, and leisure activities such as sports and socializing with friends. Various activities that individuals perceive as important in their lives are referred to as “meaningful occupations” [11]. Understanding the history, interpersonal relationships, context, and development of meaning in a client’s occupations is essential to effectively support these meaningful occupations [12]. Our scoping review of home-centered occupational therapy for children with neurodevelopmental disorders found no validated tools designed to support parental caregiving as assessed by experts for reliability and validity (K Kura, MSc, unpublished data, August 2025).

To address this gap, we developed a mobile app called the Children’s Occupation Support Mobile System (COSMO) to collaboratively support occupational therapists, children, and parents in achieving intervention goals in daily life. Recently, 71.9% occupational therapists have reported using apps as part of their clinical support, and interest is growing in expanding the use of apps specifically designed for occupational therapy [13,14]. Apps used in occupational therapy typically address areas such as education, activities of daily living, instrumental activities of daily living, and play, with the target users ranging from children to older adults [13]. Accordingly, developing and implementing COSMO as a mobile app was deemed appropriate to meet these needs. This study aimed to develop and validate the COSMO content. In addition, we outlined the basic steps of the app.

## Methods

### Study Design

This study was conducted in two stages: (1) designing a prototype of COSMO and (2) validating its content using the Delphi method. The Delphi method is a qualitative research approach commonly used to achieve expert consensus in areas such as medical guidelines and app development. It was selected for this study because it aligns with the research objectives of establishing expert agreement on the functional framework of the app and verifying its content validity. The first author (KK) was responsible for managing the Delphi process, including the study flow and questionnaire design, whereas the last author (KT) provided overall supervision and guidance. This study followed the 6-step Delphi framework recommended for health care research [15] and adhered to the ACCORD (Accurate Consensus Reporting Document) guidelines for reporting consensus-based studies [16].

### Ethical Considerations

This study complies with COPE (Committee on Publication Ethics) guidelines and adheres to the guidelines in the Helsinki Declaration. Ethics approval was obtained from the Kitasato University Ethics Review Committee (approval 2024-020). Our protocol was registered with the University Medical Information Network Center (000057084).

### Development of Prototype Apps

**Figure 1.**
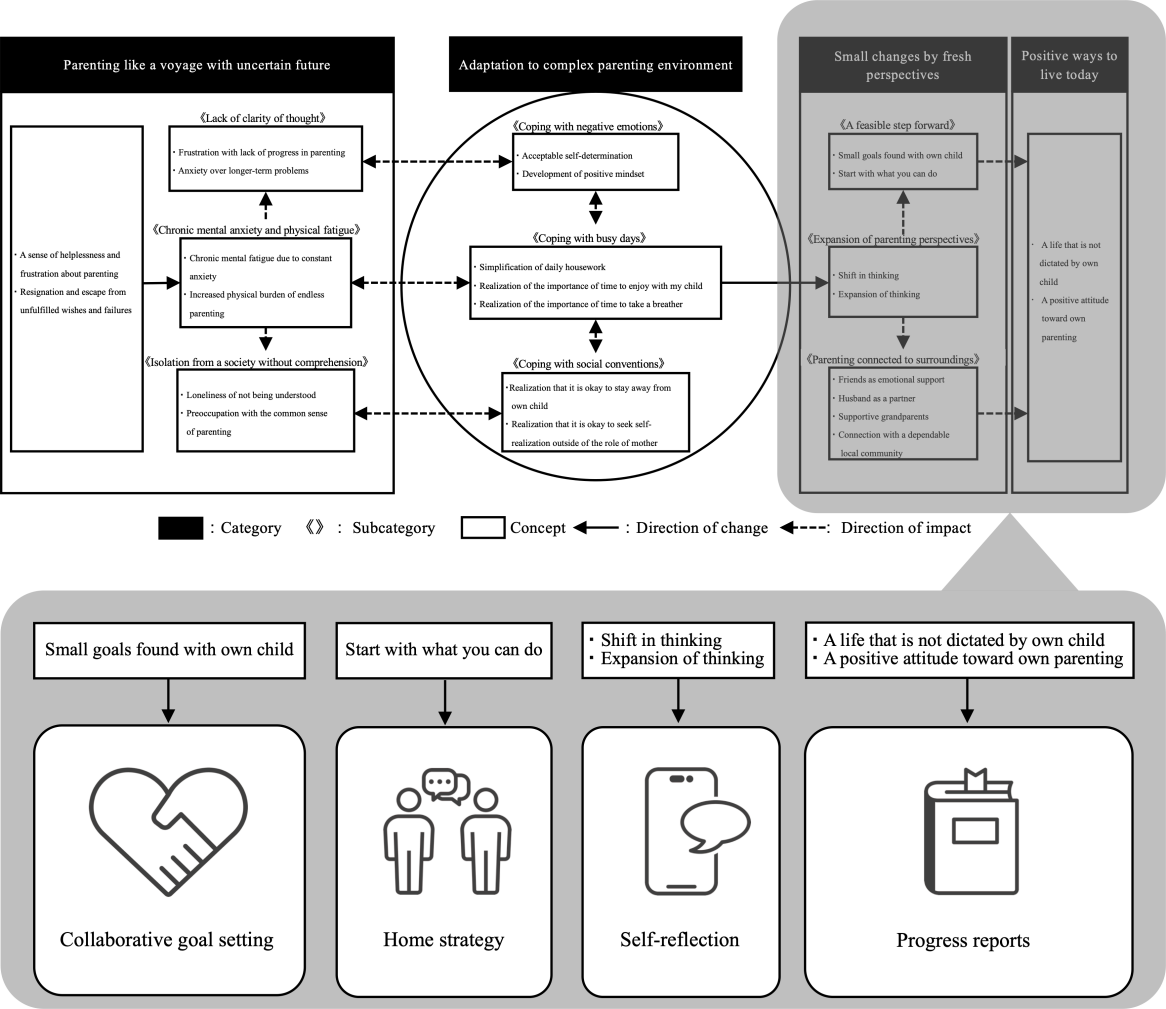
(A) Conceptual model [13] and (B) app function.

The COSMO prototype was developed based on the “conceptual model of effective parenting strategies for mothers with children who experience developmental disorders” formulated from parental perspectives and previous research [17]. The conceptual model was developed on the basis of interviews with 10 mothers who had implemented an individualized occupational therapy home program. The model illustrates the process of developing parenting strategies adapted to the home environment. The relationship between the conceptual model and the prototype app is shown in Figure 1. For example, “collaborative goal setting” corresponds to the theme “small goals found with own child,” “home strategy” corresponds to “start with what you can do,” “self-reflection” relates to “shift in thinking” and “expansion of thinking,” and “progress reports” connects to themes such as “a life that is not dictated by own child” and “a positive attitude toward own parenting.” End users were not involved in the process of deriving the prototype’s functionality from the conceptual model. The first and last authors (KK and KT) collaboratively designed the prototype through a series of 1-hour meetings held monthly, for a total of 13 meetings. Initially, COSMO’s vision was conceptually defined as “an app that supports children and families in achieving their goals within the context of parenting.” The concept of COSMO was further operationally defined as “an app that grows with the child.” It was designed to be downloaded to the parents’ smartphones and used as a tool for occupational therapy. To enhance accessibility, terminology was chosen to resonate with parents caring for their children with neurodevelopmental disorders.

The functional framework of COSMO is structured into four core components based on previous research: (1) collaborative goal setting, (2) home strategy, (3) self-reflection, and (4) progress reports.

Nine functions were developed to align with the objectives of the 4 components. The functional framework and corresponding functions were developed based on theoretical concepts and input from parents. Finally, the user interface and user experience of COSMO were designed to support these 4 functional components and 9 specific functions. The details of this framework are presented in Table 1.

**Table 1.** App function, items, and contents.

Function and item		Contents
1. Collaborative goal setting		
	Listing of goal setting	The goal-setting list function displays multiple goals on the screen, allowing users to quickly review all goals upon launching the app.
	Set time frames for achieving goals	The goal time limit function lists the deadlines for achieving each goal. By displaying the remaining time for each goal, this function clarifies the necessary steps from the current state to the goal deadline.
	Framework for goal setting by who, what, when, where, why, and how	The who, what, when, where, why, and how framework function for goal setting assists in defining specific, life-related goals using the who, what, when, where, why, and how approach. It is designed to support occupational therapists with limited clinical experience and caregivers in concretizing goals.
2. Home strategy		
	Setting multiple strategies for goals	The multiple strategy-setting function allows users to assign multiple strategies to a single goal. Strategies can be set flexibly to ensure that they are realistic and feasible.
	Notification of strategy implementation time	The strategy implementation time notification function sends smartphone notifications based on the scheduled execution time of each strategy at home. Given that the implementation timing varies by strategy, this feature ensures that each strategy is executed at the appropriate time.
	Framework for strategic planning by who, what, when, where, why, and how	The who, what, when, where, why, and how framework function for strategic planning helps formulate strategies in a structured and specific manner using the who, what, when, where, why, and how approach. It aims to facilitate the development of realistic and practical strategies.
3. Self-reflection		
	Diary recordings of reflections	Diary recordings of reflections function enables users to record the outcomes of home-implemented strategies. In addition to documenting the child’s daily care, this function supports tracking the child’s progress and provides parents with new insights.
	Caregivers’ assessment of strategies	The caregiver assessment function for strategies allows parents to evaluate the efficiency and burden of implemented strategies. This function provides a quantitative assessment of parental perspectives. The assessment timing is flexible and can be adjusted based on the occupational therapy intervention’s purpose and frequency (eg, weekly, biweekly, or monthly).
4. Progress reports		
	Goals, strategies, and record keeping	The goal, strategy, and record-saving function stores past records of goals, strategies, and outcomes. This function facilitates the reuse of effective strategies for other goals, enables tracking of home implementation, and allows for modifications to goals and strategies as needed.

### Process of Using COSMO

#### Overview

**Figure 2.**
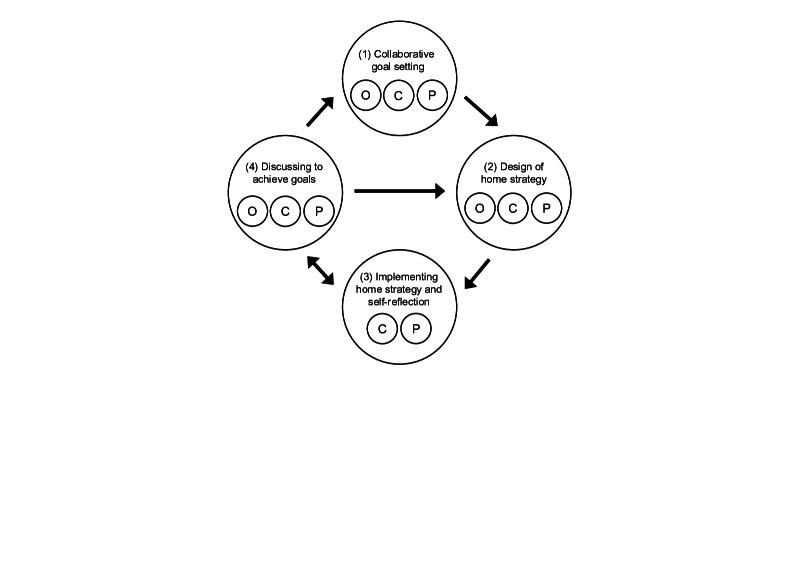
Process of using COSMO. C: child; COSMO: Children’s Occupation Support Mobile System; O: occupational therapist; P: parent.

The process flow of COSMO, based on 4 components and 9 specific functions determined through the development of the prototype app, is shown in Figure 2. The app was structured into four cyclical phases: (1) collaborative goal setting, (2) design of home strategy, (3) implementing home strategy and self-reflection, and (4) discussing to achieve goals.

#### Collaborative Goal Setting

Occupational therapists, children, and parents collaboratively established goals through shared decision-making. If the child could not actively participate, the therapist and parents set goals on their behalf. Goals were defined using the who, what, when, where, why, and how framework and adhered to the Specific, Measurable, Achievable, Relevant, and Timed (SMART) criteria whenever possible [18]. COSMO does not include built-in structured interview functions; therefore, complementary tools such as the Canadian Occupational Performance Measure [19], Goal Attainment Scaling [20], and Aid for Decision-Making in Occupation Choice (ADOC)—School Version [21] can be used alongside COSMO.

#### Home Strategy

The next step involved designing a strategy to achieve the established goals. Strategies can be linked to specific goals, and multiple strategies can be set. Intervention strategies were developed collaboratively among therapists, children, and parents. If child participation is difficult, the therapists and parents created strategies together. Each strategy was formulated using the who, what, when, where, why, and how framework to ensure that the plan is actionable and practical. Parents received smartphone notifications for each scheduled strategy.

#### Implementing Home Strategy and Self-Reflection

In the context of daily parenting, parents implemented strategies designed to help their children achieve their goals. For strategy implementation, it is important that the strategy is embedded in everyday situations and is easy to use. For example, regarding strategies related to changing clothes, this could mean changing the size of buttons so that it is easy to operate or supporting a stable posture for changing clothes. In other words, strategies that avoid placing an undue burden on parents and can be implemented as an extension of normal child-rearing practices promote daily use. Such a perspective is important in positioning parents as parental caregivers rather than cotherapists. Following implementation, a self-reflection process consisting of free-text reflection and a 5-point Likert-scale evaluation was conducted. The Likert scale assessed the child’s efficiency in executing the strategy and the level of burden experienced by the parents. Free-text reflection allowed parents to describe what has been done, identify the difficulties encountered, note observations, and determine the next steps based on the innovations they noticed. The efficiency rating measured how smoothly the child was able to carry out the strategy from the parents’ perspective, whereas the burden rating reflected the level of difficulty parents experienced in supporting the implementation.

The recorded goals, strategies, and reflections, including free text and Likert-scale outcomes, were stored as data that could be accessed at any time through the app. It is important to note that only this phase, in which the home strategy and self-reflection were implemented, was conducted independently by parents.

#### Discussing to Achieve Goals

During occupational therapy sessions, therapists reviewed progress based on the parents’ recorded reflections and data. The review process emphasized that unsuccessful strategy implementation is a natural part of progress, and therapists provided coaching and guidance accordingly. If necessary, goals and strategies could be modified by returning to phases 1 and 2. If no modifications were required, the family continued to implement the strategies until the goal was achieved. Given that pediatric occupational therapy in Japan is typically provided on an outpatient basis (every 2 weeks to once a month), COSMO is designed to complement these sessions by supporting parents between visits.

### Delphi Method for Content Validation

After developing the app prototype, content validation was conducted to assess the legibility, visibility, and accessibility of COSMO’s 4 functional frameworks and 9 functions. The COSMO is designed for use by parents with the support of pediatric occupational therapists with diverse clinical experience. Therefore, the study participants were selected using convenience sampling to ensure diversity and heterogeneity in attributes, such as age, clinical experience, educational background, and employment status.

The sample size for the Delphi method typically ranges from 5 to 20 participants [22], with no rigid rules, depending on the study’s purpose and complexity [23]. In this study, 10 Japanese pediatric occupational therapists were included based on prior research that used a similar sample size for app development in occupational therapy [24] and based on the complexity of the 4 functional frameworks and the 27-item web survey addressing 9 functions.

Participants who provided informed consent were given a 15-minute web-based briefing via a videoconferencing platform. This session covered the study background, objectives, procedures, and functions of COSMO, followed by a question and answer session to address any questions. The participants were also assured that they could withdraw from the study after receiving this explanation.

The Delphi process was conducted entirely through a web-based questionnaire (Google Forms) emailed to the experts. As the responses were collected on the web, the participants submitted their answers individually.

The questionnaire assessed the user interface or user experience design of the 9 functions within COSMO’s 4 functional frameworks, comprising a total of 27 questions. These questions evaluated 3 key aspects: legibility (clarity of text expression and display), visibility (ease of viewing text and screen layout), and accessibility (ease of functional use). Participants rated their agreement with each item on a 5-point Likert scale (1=strongly disagree and 5=strongly agree). A mean score of 3.75 (75%) or higher indicated consensus on an item [15].

To allow for qualitative feedback, the questionnaire also included open-ended questions in which participants could freely express their opinions. A summary of the results was emailed to the participants, and a detailed statistical report, including summaries of questionnaire items and responses to open-ended questions, was provided in a PDF document.

Each Delphi round had a specified response deadline. If the participant did not submit a response on time, a reminder email was sent to encourage completion.

## Results

### Overview

The demographic attributes of the 10 study participants are summarized in Table 2. The sample included 6 male and 4 female participants. In total, 4 participants were in their 20s, 4 were in their 30s, and 2 were in their 40s. In terms of educational background, 4 participants held bachelor’s degrees, 3 held master’s degrees, and 3 held doctoral degrees. The occupational therapists had an average of 7.9 (SD 3.2; range 2-12) years of clinical experience.

**Table 2.** Participant demographics.

Participants	Sex	Age (years)	Employment	Degree	Time in profession (years)
A	Male	43	Regular	PhD	12
B	Female	28	Nonregular	Master	7
C	Male	27	Regular	Master	2
D	Male	29	Regular	Bachelor	6
E	Male	33	Nonregular	Master	9
F	Female	30	Regular	Bachelor	9
G	Female	41	Regular	Bachelor	12
H	Male	31	Regular	PhD	9
I	Male	32	Regular	Bachelor	10
J	Female	29	Regular	PhD	3

### Delphi Method Results

In the first round of the Delphi method, the mean score for all 27 items exceeded the consensus threshold of 3.75, with an overall average of 4.50. Research participants suggested modifying the language to make it easier for the intended users to understand and making input items optional to reduce the burden of use. These comments were reviewed and addressed by the research team, considering the degree of variation in participant feedback and outcomes of team discussions. Based on feedback from the open-ended responses, modifications were made to the app design, and a second round was conducted using the revised questionnaire. In this round, the average score increased to 4.84. Across all 27 items (comprising 9 items each for legibility, visibility, and accessibility), the mean scores for each evaluation category increased by 0.20 to 0.45 points between rounds. Furthermore, the IQR was reduced from 0.00‐2.00 in the first round to 0.00‐1.00 in the second round, indicating a decreased level of response variability. Because only minor revisions were suggested in the free-text comments, these were incorporated, and the Delphi process was concluded. The results of each round are presented in Table 3. Detailed comments from study participants and corresponding responses from the research team are presented in Multimedia Appendix 1. The findings are described below according to 3 evaluation criteria: legibility, visibility, and accessibility.

**Table 3.** Results of the first and second rounds of the Delphi method (n=10).

Function and item		First round						Second round					
		Legibility		Visibility		Accessibility		Legibility		Visibility		Accessibility	
		Mean (SD)	Median (IQR)	Mean (SD)	Median (IQR)	Mean (SD)	Median (IQR)	Mean (SD)	Median (IQR)	Mean (SD)	Median (IQR)	Mean (SD)	Median (IQR)
1. Collaborative goal setting													
	Listing of goal setting	4.80 (0.40)	5.00 (5-5)	4.80 (0.40)	5.00 (5-5)	4.50 (0.67)	5.00 (4-5)	5.00 (0.00)	5.00 (5-5)	5.00 (0.00)	5.00 (5-5)	4.90 (0.30)	5.00 (5-5)
	Set time frames for achieving goals	4.20 (0.98)	5.00 (3-5)	4.40 (0.80)	5.00 (4-5)	4.40 (0.80)	5.00 (4-5)	5.00 (0.00)	5.00 (5-5)	5.00 (0.00)	5.00 (5-5)	4.90 (0.30)	5.00 (5-5)
	Framework for goal setting by who, what, when, where, why, and how	3.90 (0.70)	4.00 (3.25-4)	4.70 (0.46)	5.00 (4.25-5)	4.40 (0.80)	5.00 (4-5)	4.70 (0.64)	5.00 (5-5)	4.80 (0.40)	5.00 (5-5)	4.90 (0.30)	5.00 (5-5)
2. Home strategy													
	Setting multiple strategies for goals	4.60 (0.92)	5.00 (5-5)	4.70 (0.46)	5.00 (4.25-5)	4.60 (0.66)	5.00 (4.25-5)	5.00 (0.00)	5.00 (5-5)	4.80 (0.40)	5.00 (5-5)	4.80 (0.40)	5.00 (5-5)
	Notification of strategy implementation time	4.80 (0.40)	5.00 (5-5)	4.70 (0.46)	5.00 (4.25-5)	4.40 (0.80)	5.00 (4-5)	4.80 (0.40)	5.00 (5-5)	4.90 (0.30)	5.00 (5-5)	4.70 (0.46)	5.00 (4.25-5)
	Framework for strategic planning by who, what, when, where, why, and how	4.10 (0.83)	4.00 (3.25-5)	4.50 (0.67)	5.00 (4-5)	4.40 (0.80)	5.00 (4-5)	4.70 (0.64)	5.00 (5-5)	4.90 (0.30)	5.00 (5-5)	4.80 (0.40)	5.00 (5-5)
3. Self-reflection													
	Diary recordings of reflections	4.50 (0.92)	5.00 (4.25-5)	4.60 (0.49)	5.00 (4-5)	4.00 (0.89)	4.00 (3-5)	4.80 (0.40)	5.00 (5-5)	4.70 (0.46)	5.00 (4.25-5)	4.80 (0.40)	5.00 (5-5)
	Caregivers’ assessment of strategies	4.50 (0.81)	5.00 (4.25-5)	4.90 (0.30)	5.00 (5-5)	4.40 (0.66)	4.50 (4-5)	4.60 (0.49)	5.00 (4-5)	4.80 (0.40)	5.00 (5-5)	4.90 (0.30)	5.00 (5-5)
4. Progress reports													
	Goals, strategies, and record keeping	4.60 (0.66)	5.00 (4.25-5)	4.60 (0.66)	5.00 (4.25-5)	4.40 (0.80)	5.00 (4-5)	4.80 (0.40)	5.00 (5-5)	4.80 (0.40)	5.00 (5-5)	4.90 (0.30)	5.00 (5-5)
Overall score		4.44 (0.82)	5.00 (4-5)	4.66 (0.56)	5.00 (4-5)	4.39 (0.78)	5.00 (4-5)	4.82 (0.44)	5.00 (5-5)	4.86 (0.35)	5.00 (5-5)	4.84 (0.36)	5.00 (5-5)

### Legibility

The results of the first round showed that the average score for all 9 items was 4.44. Regarding legibility, the participants suggested modifying the wording and standardizing expressions. Specifically, on the goal-setting screen, the phrase “the occupational activity you want to be able to do” was changed to “what (the activity you want to be able to do).” This adjustment avoided the use of the technical term “occupational activity” and adopted language that was easier for parents to understand. Additionally, “what” was added at the beginning of the sentence to align with the who, what, when, where, why, and how framework. There was a suggestion to change “deadline” in the goal list screen to “duration.” However, this modification was not implemented because “deadline” aligns with the time-related component of the SMART criteria in goal setting. On the strategy-setting screen, “what” was added to “in what way” to ensure consistency. In the self-reflection efficiency and burden evaluation screen, the subject of evaluation was clarified by modifying the phrasing to “How smooth was this strategy for the child?” and “How easy was this strategy for the parent or guardian?” These changes were made to enhance clarity.

The results of the second round showed that the average score for all 9 items increased to 4.82. Additionally, the terminology used to describe guardians was revised, as the participants noted that the original wording was unfamiliar to the general public in Japanese.

### Visibility

The results of the first round showed that the average score for all 9 items was 4.66. Regarding visibility, the participants suggested improving the consistency of screen displays. Specifically, there was a recommendation to unify goal- and strategy-setting screens, given that the goal-setting screen provides a free-description framework for each who, what, when, where, why, and how component, whereas the strategy-setting screen lacks this feature. After discussion, the research team decided not to implement this change because the 2 screens serve different purposes. The goal-setting screen includes a who, what, when, where, why, and how framework to facilitate goal setting based on the SMART criteria, whereas the strategy-setting screen allows flexible descriptions, as the content of strategies may vary among households. This reasoning was explained to the participants during feedback. There was also a request to include example sentences on the strategy-setting screen. However, because of screen space limitations and the number of characters displayed, this suggestion was not implemented. Instead, example sentences were included in the user guide under development.

The results of the second round showed that the average score for all 9 items increased to 4.86. In addition, the participants suggested increasing the font size for better readability. Based on this feedback, the text size was adjusted to improve the readability for children and parents.

### Accessibility

The results of the first round showed that the average score for all 9 items was 4.39. Regarding accessibility, participants raised concerns about the difficulty of setting goals using the who, what, when, where, why, and how framework, the burden of free-text input in self-reflection, the sequence of outcome assessments, and the need for additional data storage options. One concern was the difficulty in completing all the who, what, when, where, why, and how components on the goal-setting screen. Based on participant feedback, each who, what, when, where, why, and how framework was made optional, allowing goal setting to be completed even if some components were left blank. To reduce participant burden, free-text input and outcome evaluations were made optional, ensuring that the data could still be stored even if only one of these components was completed. The participants also suggested reordering the self-reflection process by placing free-text reflections after the outcome evaluation of efficiency and burden. However, the research team decided not to implement this change because verbalizing and reflecting on the strategies in the free-response section first was considered beneficial for conducting a structured 5-stage evaluation. Regarding data storage, there was a recommendation to store efficiency and burden evaluations along with other data. This suggestion was accepted, and these evaluations were added to the list of stored items.

The results of the second round showed that the average score for all 9 items increased to 4.84. No further modifications were made, as there were no significant concerns raised by the participants.

## Discussion

### Principal Findings

In this study, the content validity of COSMO was evaluated using the Delphi method. Consequently, COSMO was designed based on expert consensus regarding legibility, visibility, and accessibility in app design. Additionally, the process of using the developed COSMO was presented. Based on these findings, this section discusses the design of COSMO, its clinical applications, and usability.

### Design of COSMO

COSMO primarily aims to support children and their families in accomplishing important tasks. More specifically, it is designed to assist parents in their daily parenting activities through practical interventions. To fulfill this purpose, COSMO was developed as a smartphone app that parents could use as part of their professional support. The coaching approach in rehabilitation emphasizes real-life activities, goal setting related to participation in daily situations, and improvements in quality of life [25]. Additionally, identifying small achievable goals with one’s child and starting with what is feasible are essential steps toward reaching broader objectives [17]. COSMO facilitates goal setting through a who, what, when, where, why, and how framework, reflecting the perspectives of parents and children while ensuring that goals align with the SMART criteria [18]. The strategy-setting function allows users to develop action plans tailored to their individual goals by specifying the number of steps and content necessary to achieve them. Therefore, COSMO includes the core functionalities required for collaborative goal setting and strategy development in daily life.

Strategy implementation in real-life settings and self-reflection play crucial roles in goal attainment. A randomized controlled trial of family-centered parent-coaching interventions demonstrated that reflecting on a child’s thoughts, motivations, and feelings enhanced parental self-evaluation and awareness [26]. In COSMO, self-reflection is facilitated through free-text input and a 5-point Likert scale. Free-text reflections encourage parents to verbalize their perceptions of strategy implementation, which may increase their awareness of goal progress. The Likert scale assessed 2 aspects: the efficiency of the child’s actions within the strategy and the burden experienced by parents. By quantitatively measuring these aspects, COSMO helps to visualize the effectiveness of strategies and enables further refinement for improved usability.

### Clinical Applications of COSMO

COSMO is expected to function as a support tool for home-centered occupational therapy. Systematic reviews of pediatric occupational therapy interventions emphasize the importance of supporting parental engagement in home settings, with collaboration between occupational therapists and parents as a key factor [3]. Existing tools used in occupational therapy include booklets outlining intervention objectives and policies [10], documents for recording strategies [8], and structured diaries for tracking daily intervention progress [9]. COSMO integrates multiple functions including goal setting, strategy development, reflection, and data storage, making it a comprehensive tool that can be flexibly applied in various intervention settings. However, careful consideration is necessary when determining whether the COSMO is appropriate for a given intervention context. A qualitative study conducted by the first author with mothers who practiced home programs suggested that using home programs and daily diaries may contribute to a positive reassessment of children’s behavior and parental burden [17]. The comprehensive functions of COSMO may influence parents’ perceptions of their children and themselves, motivating them toward goal achievement.

Finally, the Delphi method assessment of accessibility yielded recommendations for reducing the parental burden. Based on these recommendations, free-text reflections and Likert-scale assessments were made optional to accommodate varying levels of parental engagement. Occupational Performance Coaching, a widely used support approach in occupational therapy, sets exclusion criteria for parents with low physical or mental energy [27]. In line with this principle, COSMO was designed to minimize the parental burden wherever possible. However, some level of effort is still required for its effective use. Therefore, the decision to use COSMO should consider the physical and mental conditions of parents or guardians. Moreover, because the COSMO is only a support tool, its implementation should be assessed within the broader context of the intervention objectives, treatment plans, and social background of the target population.

If adverse effects emerge after implementation and continued use poses risks, the occupational therapist in charge should consider discontinuing its use.

### Usability of COSMO

Usability for professionals and clients is essential to ensure that apps are accessible and effective. Previous studies have reported that the main purposes of using apps in occupational therapy are to enhance clients’ skills, facilitate participation in daily activities, and support the therapeutic process [14]. Furthermore, apps should be matched to client needs, skills, and abilities and not the other way around [28]. The selective use of apps by occupational therapists based on individual client needs suggests that, as with other therapeutic interventions, such tools should not be uniformly applied to all clients [14,28]. Similarly, the implementation of COSMO requires the careful consideration of the intervention’s goals and the unique needs of each client.

Several barriers to app usability among professionals have been identified. Prior studies have cited the most common reasons for not using apps as the inability to find suitable good apps, never thought about it, and lack of access [14]. The first 2 barriers may also apply to COSMO, highlighting the importance of building further evidence and promoting broader awareness. To address accessibility, COSMO will initially be distributed free of charge in Japan. For households without smartphones, a paper-based version offering equivalent functions will be made available via the project website. Because COSMO is intended as a supportive tool for daily life rather than a treatment device, it complies with medical app review requirements. Moreover, because COSMO is not a research app, it does not collect any personal client information via mobile networks. The app is downloaded and stored on the client’s personal smartphone, minimizing the risk of data disclosure to third parties. Thus, potential concerns about data protection have been addressed.

Currently, app use in occupational therapy in Japan is generally regarded as a supplement to standard treatment. Therefore, COSMO—being freely accessible—is expected to complement existing occupational therapy and other therapeutic interventions based on professional clinical reasoning. In Japan, several specialized apps have been developed by occupational therapists, such as Osca Writing, a Japanese handwriting app [29], and Process Analysis of Daily Activities for Dementia, which analyzes daily occupations for individuals with dementia [30]. Additionally, goal-setting apps like ADOC and ADOC—School Version have been introduced [21,31]. Although the first 2 differ from COSMO in purpose and target populations, ADOC shares some similarities with COSMO in terms of goal setting. However, ADOC focuses on shared decision-making in goal selection, whereas COSMO emphasizes goal-sharing and achievement processes. Although this study was limited to comparisons with domestic applications due to differences in social and health care contexts, future studies should include comparisons across diverse health care systems and cultures.

### Limitations

This study has some limitations. First, end-user children and parents were not included in the development process of this study; this is a significant limitation. Future usability tests should be conducted with the involved parties. Second, the Delphi method involved participants from a limited number of regions, which may have influenced the development of COSMO because of the homogeneous educational and cultural backgrounds of the participants. Third, convenience sampling—where participants were recruited through the researchers’ networks—was conducted to ensure diversity and heterogeneity among participants and to enhance research feasibility; however, this limits the generalizability of the findings and may have influenced the response outcomes. Fourth, the quality of COSMO as a mobile app is currently unknown. The assessment of app quality is important for its future adoption by clients and professionals. Fifth, only the Japanese version of COSMO has been developed. Future studies should examine the cross-cultural validity, including translation accuracy and adaptation, for use in other countries. Sixth, the social implementation of COSMO was initially developed for use in Japan. Consequently, the current discussion does not sufficiently address the challenges of implementation in diverse health care settings.

### Conclusions

COSMO was developed to support children and their families in achieving meaningful occupations. After 2 rounds of the Delphi method evaluation, the content validity of COSMO in terms of legibility, visibility, and accessibility was confirmed through an expert consensus. As a home-centered occupational therapy support tool, COSMO is expected to be flexibly integrated into interventions, depending on the objectives of the therapy. To optimize its use, discussions among professionals, parents, and children are essential before recommending its implementation. Ultimately, COSMO has the potential to enhance parental participation in achieving children’s goals and contribute to more effective occupational therapy interventions. A dedicated website has been created, including a contact page to provide ongoing technical support. Because the app was developed for iOS, updates will be made as necessary to address technical issues arising from iOS system changes. Future studies should explore cross-cultural validity, including translation accuracy and adaptation, and should assess generalizability and effectiveness as an intervention tool.

## Supplementary material

10.2196/73430 Multimedia Appendix 1 Comments of the Delphi methods in 2 rounds.

## Abbreviations

ACCORD: Accurate Consensus Reporting Document
ADOC: Aid for Decision-Making in Occupation Choice
COPE: Committee on Publication Ethics
COSMO: Children’s Occupation Support Mobile System
SMART: Specific, Measurable, Achievable, Relevant, and Timed
