# Supplementary material for: Development of Goal-Achievement Support App to Assist Children and Families in Participating in Meaningful Occupations: Content Validation Using Delphi Method
Source: JMIR Rehabil Assist Technol. 2025 Aug 20;12:e73430. doi: 10.2196/73430 (PMC12367348; doi:10.2196/73430)
Supplement: Multimedia Appendix 1 [file rehab-v12-e73430-s001.docx]

1. First Round

| Function | Comments | Response |
| --- | --- | --- |
| 1. Collaborative Goal Setting | I thought that some users might find it difficult to answer the "where" and "how" components. | We have followed your suggestion and modified the text so that it will work as a sentence in the application even if it is left blank. |
|  | I felt somewhat uncomfortable with the expression “occupational activities you want to be able to do.” The other items are described more generally, so this phrase stood out. I believe the term “occupational activities” may be difficult for typical parents to understand. | Accordingly, we have revised the wording to: “what (the activity you want to be able to do).” |
|  | I think it would be more user-friendly if the goal-setting screen allowed for input using dropdown menus or similar tools. | After discussion with our research team, we decided to retain the current use of open-ended responses. This allows for individualized input, and no other participants raised similar concerns. |
|  | Deadlines make me anxious. Sometimes the goals or the staged goals don’t seem achievable, so I wonder if it could instead be a moment to reflect, rather than a strict deadline. | Following team discussion, we have decided to maintain the current use of time-related expressions (i.e., clear deadlines), as recommended in the SMART criteria. Given that no similar suggestions were made by other participants, we opted not to revise this aspect. However, in actual clinical use, we would recommend that occupational therapists (OTs) apply the concept flexibly—either as a deadline or as a reflection period—depending on the intervention’s purpose. |
|  | The text is easy to read. However, it may feel too simple to be engaging—especially for parents raising children. I think it would be more appealing with the addition of icons or illustrations rather than text alone. | As this study focused solely on evaluating legibility, visibility, and usability, it is beyond the current scope to implement design elements such as illustrations. However, we will consider incorporating icons and illustrations during the full-scale development phase, as appropriate. |
|  | Regarding the goal-setting framework wording: the top four items follow the 5W1H format, but the final item, “occupational activities you want to be able to do,” does not. It might be clearer and more consistent if it were phrased as “what (occupational activity you want to be able to do).” | Accordingly, we have revised the wording to: “what (the activity you want to be able to do).” |
|  | I found the “how” component of the sentence difficult to understand. It would be helpful if example sentences could be shown as annotations when needed. The phrase “occupational activities” was also hard to grasp. I think simpler wording like “what I want to be able to do” would be easier to understand. The usability would also improve if example sentences were provided. | We have revised the expression “work activity” to “what (the activity you want to be able to do).,” as you suggested.  Regarding the inclusion of example sentences, we decided to retain the current format after team discussion, given that few similar requests were made by other participants and we aimed to reduce visual complexity on the app interface. However, because goal-setting will be conducted collaboratively with OTs, we plan to include example sentences in the user manual or guidance materials to support meaningful suggestions by therapists. |
|  | I feel that the terms “deadline” or “period” might be interpreted differently from the intended “time frame” for goal achievement. I also think that the meaning of “occupational activities” may differ between occupational therapists and guardians. I was wondering if it could be as roughly worded as “what I would like to be able to do.” | As a result of internal discussions, we will continue using the expression “time-related (clear deadline)” in line with SMART criteria. If interpretation varies depending on the intervention purpose, we suggest that OTs clarify whether the “deadline” represents a specific date or a general timeframe during the informed consent process.  We have revised the expression “work activity” to “what (the activity you want to be able to do).,” as you suggested. |
| 1. Home Strategy | It was unclear whether the notification function implies are everyday frequency. | At this stage, we ask that the notification function be understood as “a feature that sends notifications to the user’s smartphone.” Specific notification settings will be discussed during the app development phase. |
|  | It depends on the user, but I thought it would be easier to fill out the form if it aligned with 5W1H items like the goal format. | As discussed by our team, we would like to ensure flexibility in the strategy-setting section—unlike goal-setting—allowing users to include specific techniques or brief remarks such as “use a timer at the beginning of the activity” or “the father will call out to the child.” |
|  | I think the 5W1H free-text feature is a great idea. The notification function is also helpful for busy parents. Some might prefer to receive reminders 10 minutes before and 10 minutes after the scheduled time. It would be great if parents could customize both the timing and frequency of notifications. | Currently, we would appreciate it if you could regard notifications as a “function that brings notifications to your phone”.  We will discuss the settings during actual development. |
|  | It may be clearer to describe “in what way” using the phrase “what and how.” | According to your suggestion, on the strategy-setting screen, "what" was added to "in what way" to ensure consistency. |
|  | I wonder whether the term “strategy” would be easily understood by parents. It might be helpful to either annotate it or consider using simpler alternatives like “home task”. There may be many activities that are not done every day, as in the text example, it would be better if the app could be customized—for instance, silent notifications or choosing which days of the week to receive them. Also, the 5W1H structure used in the strategy section should be unified with the goal-setting framework. I would also appreciate the inclusion of example sentences. | After discussion within the research team, we decided not to change the term “strategy” to “homework,” as no similar requests were raised by other participants, and “homework” may imply externally assigned tasks. However, we agree that this term can be explained flexibly depending on the OT’s purpose during implementation.  As noted above, we aim to preserve flexibility in strategy input by allowing brief practical notes in addition to specific actions. |
|  | Although I don't think this needs to be addressed immediately, I felt that the simplicity and flexibility of the display could be highly effective for strategic planning—particularly when the child is in middle school and the parents have come to terms with the disability. On the other hand, for families with younger children or parents who are still in the early stages of acceptance, this approach might feel somewhat burdensome. I believe the necessity of this method will become clearer depending on the characteristics of the intended user group, so specifying the target of caregiver users more clearly in the future may help. | As you suggested, more specific configuration options would be useful for parents of elementary and middle school children. However, as a foundational design principle, this app is intended to be used in parallel with occupational therapy sessions. Therefore, for families with younger children or for parents who may struggle with setup, we expect that OTs will provide guidance, working collaboratively to develop appropriate strategies.  Regarding the intended users, we recognize that this tool may not be feasible for families experiencing significant physical or mental fatigue. This point will be taken into account in future discussions on user targeting and support. |
| 1. Self-Reflection | I felt that it would be easier to answer the question if it asked about the user’s experience after the star rating. | After discussion within the research team, we decided to maintain the current workflow. This decision was based on the fact that another participant expressed support for the existing structure, and we believe that verbalizing reflections, reviewing them, and then conducting a quantitative evaluation helps to clarify key aspects of performance. |
|  | Writing should be done on special notes or when there is time, and it would be good to be able to look back easily. | In response to your suggestion, we have modified the system so that free-text entries are recorded even if left blank or only include special remarks. |
|  | Is the record achievement-based? It might be easier for parents to first complete a simple checklist, such as circles (○), crosses (×), or triangles (△), or use a star rating (☆) on the next page, before entering a free-text description. I also feel that the phrase “degree of efficiency” could be confusing for parents—it may be interpreted in terms of how smoothly they were able to support their child. | As mentioned above, the research team discussed this issue but decided to retain the current workflow, as only one other participant expressed a similar concern. We believe that the process of verbalizing reflections, reviewing them, and subsequently conducting a quantitative evaluation helps clarify the key points to be assessed.  To enhance clarity, the outcome evaluation items have been revised to specify the subjects as follows:  “How smooth was this strategy for the child? (efficiency)”  “How easy was this strategy for the parent or guardian? (burden)” |
|  | I would appreciate example entries for the free-text descriptions, as the current format feels very open-ended. It would also be helpful to have selectable options when the strategy could not be carried out (e.g., ◯ or ×). For busy parents, simply marking ◯ or × might be more feasible. I also found the subject of the outcome section difficult to understand—especially whether “burden” refers to the child’s burden or the caregiver’s burden. | Following internal discussion, we decided that example sentences would not be included directly on the application screen (including for the strategy section), but would instead be provided in the user manual or guide.  To accommodate cases in which a strategy could not be implemented, we revised the input form to allow outcome evaluations to be recorded even if the free-text section is left blank.  Additionally, completing a retrospective entry is not mandatory, and it is acceptable for users to skip this on days when the notification is not acted upon.  The subject of the outcome evaluation was modified as described in the previous response. |
| 1. Progress Reports | I thought it would be nice to be able to record a look back at the involvement that was good or at least when it was not good enough. | If the primary aim of the occupational therapy intervention is to reflect on successful and unsuccessful aspects of the caregiver’s involvement, we suggest that client be instructed to provide free-text descriptions only when such reflections are meaningful. |
|  | I wanted to see how the screen would change if there were multiple goals. I thought it would be motivating if there was something that would allow parents and children to check their efforts when looking back. I imagine it would be like a graph of weight gain or loss when dieting. | In line with your suggestion, we have added a star-based rating system to the saved outcome items.  Given that the app does not currently include a function to rate goal attainment directly, we anticipate that formal reassessment will be conducted using validated tools such as the Canadian Occupational Performance Measure (COPM) or Goal Attainment Scaling (GAS). We appreciate your valuable input on this matter. |
|  | It would be nice if the outcome evaluations could be stored in a visually pleasing and easy-to-understand manner. | As suggested, we have added outcome evaluation results to the saved items. |
|  | I felt that it would be easier to understand at a quick glance if there was also an outcome evaluation in the data storage. | As suggested, we have added outcome evaluation results to the saved items. |

1. Second Round

| Function | Comments | Response |
| --- | --- | --- |
| 1. Collaborative Goal Setting | It is good that the system allows entries even if some fields are left blank, but I think certain restrictions can help prompt more specific thinking. I found it difficult to strike the right balance. Providing a help function or guidance to encourage concrete responses might be important. That said, it depends on how the tool is used—so it’s hard to say definitively. | This feature is primarily intended to be configured collaboratively with occupational therapists during the course of therapy.  Therefore, we kindly ask that clinical judgment be used to determine whether to set limits or allow flexibility, depending on the specific objectives of the occupational therapy intervention. |
|  | We felt that many parents would find the tool easier to use if font size adjustment were available in the commercial version. | Thank you for your suggestion.  We will take it into consideration during the development process. |
|  | I felt that ending the phrase with “what” was somewhat awkward. It might be clearer and more natural to conclude with a verb, such as in “I want to be able to do”. | After discussion within the research team, and in the absence of similar suggestions from other participants, we have decided to retain the current implementation. |
| 1. Home Strategy | I thought imagination might be easier if there were descriptive examples available in the help section. Well-crafted goals and strategies can inspire other parents. | Thank you for your suggestion.  We currently plan to include examples of goals and strategies in the user manual. |
|  | I think it would be easier to complete the form if example sentences were included. | As noted above, examples of goals and strategies are planned to be provided in the user manual. |
|  | I found that the update made the input process easier. I also appreciate the reminder feature. | Thank you for your valuable feedback. |
|  | It would be nice if the notification time could also be left blank. | Following discussion within the research team and given the lack of similar proposals from other participants, we have decided to maintain the current design. |
| 1. Self-Reflection | While I think it should be possible to opt out of journal entries during reflection, the primary goal of the application is to encourage that reflection. Therefore, it may be more effective if the app design gently encourages users to write journal entries—for example, by showing sample entries in the help section or explaining the benefits of journaling. I agree with the concept, but I believe the feature could be improved. | We believe that self-reflection is a fundamental component of occupational therapy.  At the same time, introducing this application to caregivers who are already overwhelmed and unable to reflect may not be appropriate.  Therefore, we believe it is essential that occupational therapists provide adequate explanation regarding the importance of evaluation and reflection, based on evidence-based practice (EBP), before introducing the application.  We also plan to include sample entries in the user manual. |
|  | The burden and efficiency ratings seem to be reversed (more stars for higher efficiency, fewer stars for lower burden), which made it slightly confusing to interpret at a glance. With closer inspection, though, it is understandable. | The item in question is designed to assess the caregiver's sense of "comfort."  We hope this can be interpreted such that a higher score reflects an increased sense of comfort. |
|  | I think it’s good that journal entries are optional, as it makes the app easier to use. The star rating system is intuitive and easy to understand. | Thank you for your valuable input. |
|  | Therapists use the term “caregiver,” but I think some family members might not understand this term clearly. It might be more user-friendly to display it as “caregiver (guardian)” or “guardian (family member).” | Following internal discussion, we have revised the term “caregiver” to “guardian.”  ※The Japanese term originally used for “parent” was replaced with a more easily understood expression for the intended users. |
| 1. Progress Reports | Recently, iPhones have a healthcare feature that shows changes in mental health over time. I thought it would be helpful to include a similar feature in this app—something that visualizes changes in task execution and satisfaction levels over time. That said, I recognize that having too many features can reduce usability, so this is just a suggestion. | Thank you for your insightful feedback.  We agree that the ability to visualize progress over time may help motivate clients.  We will consider incorporating this feature in future updates. |
|  | I understood that more stars indicate less burden, but if clarity is a priority, a visual symbol like a smiley face might be more intuitive. | Thank you for your suggestion regarding visual clarity.  Although no similar feedback was received from other participants, we will keep your comment in mind for future revisions, while maintaining the current design at this stage. |
|  | I tend to understand things more effectively through visual information, so the update improved how quickly I could process what I was seeing. Mothers often experience cognitive fatigue due to the multitasking demands of parenting, so I feel it is quite important that this can be implemented without added strain. | Thank you for your valuable feedback.  We hope that this tool will contribute to reducing the burden on parents and support the provision of empowering occupational therapy in the context of parenting. |
